# DNA barcoding of the supergiant isopods from *Bathynomuskensleyi* Lowry & Dempsey, 2006 (Cirolanidae) and a molecular biology comparison of *B.jamesi* Kou, Chen & Li, 2017

**DOI:** 10.3897/BDJ.12.e111046

**Published:** 2024-01-05

**Authors:** Ming-Chih Huang, Niel L Bruce

**Affiliations:** 1 Department of Biological Sciences and Technology, National University of Tainan, Tainan City, 700-301, Taiwan Department of Biological Sciences and Technology, National University of Tainan Tainan City, 700-301 Taiwan; 2 Water Research Group, Unit for Environmental Sciences and Management, North-West University, Private Bag X6001, Potchefstroom 2520, South Africa Water Research Group, Unit for Environmental Sciences and Management, North-West University, Private Bag X6001 Potchefstroom 2520 South Africa; 3 Biodiversity and Geosciences Program, Queensland Museum, PO Box: 3300,, South Brisbane BC, Queensland 4101, Australia Biodiversity and Geosciences Program, Queensland Museum, PO Box: 3300, South Brisbane BC, Queensland 4101 Australia

**Keywords:** COI, DNA sequence, *Bathynomus*, Cirolanidae, Isopoda, South China Sea, Indian Ocean, India

## Abstract

DNA was extracted from tissue samples from specimens of newly-collected *Bathynomuskensleyi* from Queensland and subsequently the COI and 16S rRNA sequences were successfully cloned. The holotype of *B.kensleyi* was also sampled for COI only. Comparison of the sequences showed that, for the COI sequences, *B.jamesi* and *B.kensleyi* have more than 59 different DNA positions amongst 596 known reading sequences. The Kimura two parameter (K2P) distance analysis confirmed that *B.jamesi* and *B.kensleyi* are two species. Indian records of *Bathynomus* are reviewed and three of the four identified species from India are shown to be misidentifications. *Bathynomusdecemspinosus*, *B.doederlini* and *B.kensleyi* are found to not occur in India and the only accepted record is that of *Bathynomuskeablei* Lowry & Dempsey, 2006. We conclude that, based on molecular analysis and morphological comparisons, the correct species identity of Indian species other than *Bathynomuskeablei* remains unknown.

## Introduction

Giant isopods of the genus *Bathynomus* Milne-Edwards, 1879, appear to many people as mysterious alien fantasy creatures, captivating them with their strange and heavily armoured appearance. *Bathynomus* occurs at depths from as little as 100 m to more than 2000 m and, as such, these deep-water isopods are rarely directly seen by humans. Species of *Bathynomus* are known to be deep-sea benthic scavengers, feeding on the remains of various organisms that have fallen from the upper layers of the ocean to the bottom of the sea (Britton and Morton 1994). These debris are mainly fish, cephalopods and decapods (Briones-Fourzán and Lozano-Alvarez 1991, Barradas-Ortiz et al. 2003), but also whale carcasses (Onishi et al. 2018). In addition, *Bathynomus* may also prey on other deep-sea organisms (Shih 1972, Briones-Fourzán and Lozano-Alvarez 1991). The widespread ‘popular’ appeal of *Bathynomus* has been instrumental in increasing human awareness of the deep-sea environment.

**Background**

The genus *Bathynomus* and its then sole species *Bathynomusgiganteus* Milne-Edwards, 1879 garnered high interest (Holthuis and Mikulka 1972) in the years following its discovery. At that time, the perceived extremely large size of the species, in comparison to other marine isopods, was an outstanding character. Very shortly after being described, *B.giganteus* was again recorded from the Western Atlantic, but also repeatedly from the northern Indian Ocean, at localities off Yemen, Goa, Kerala, Tamil Nadu and off Myanmar (Holthuis and Mikulka 1972). Identifying authors never questioned that their ‘giant’ isopods might be different to the Atlantic species. These multiple records led to the acceptance by more recent workers that *Bathynomusgiganteus* occurred in the northern Indian Ocean and led to further records from India (Lyla et al. 2007, Nayak et al. 2007) and China (Soong 1992). Lowry and Dempsey (2006), re-examining the available specimens, clearly established that all records from the Indian Ocean were misidentifications of *Bathynomusgiganteus* and described the new species *Bathynomuskeablei* Lowry & Dempsey, 2006 and, from Australia and the South China Sea, *Bathynomuskensleyi* (Lowry and Dempsey (2006)).

Character interpretation and understanding of *Bathynomus* has increased over time, but it is only recently that it has been realised that, on both morphological criteria and, critically, also genetic criteria, there are cryptic species within the genus *Bathynomus*. The large size and physical uniformity of appearance have misled many trying to identify species, including experts. In that regard, *Bathynomus* is no different to other cirolanid genera in that groups of morphological ‘cryptic’ species, such as the *Cirolana* ‘*parva* group’ (Bruce 2004, Rodcharoen et al. 2016, Sidabalok and Bruce 2017) are commonplace. This has most recently been highlighted by the recognition of the western Atlantic ‘supergiant’ *Bathynomusyucatanensis* Huang Kawai and Bruce, 2022, initially detected through molecular analysis and, morphologically, almost identical to *Bathynomusgiganteus*. At the same time, these authors also showed that the original records of *Bathynomuskensleyi* were not all of one species, but rather included two other effectively cryptic species, one of these later described as *Bathynomusjamesi* Kou, Chen & Li, 2017 with an effective distribution within the South China Sea and the third being an undescribed deep-water species from the eastern Philippines (Huang et al. 2022). The species of *Bathynomus* from the Spratly Islands, identified as *B.kensleyi* by Truong (2015), remains unresolved. Sidabalok et al. (2020) also noted that specimens from the Arafura Sea, identified as *Bathynomusaffinis* by Lowry and Dempsey (2006), could not, in fact, be that species as there were some clear differences in uropod morphology, suggesting again that cryptic species could exist within this group of *Bathynomus* species. Of the 20 extant species of *Bathynomus*, nine belong to the 'supergiant' species (ranging in size from 150 mm to 500 mm) and 11 species are 'giant' species (that range in size from 90 to 140 mm) (Lowry and Dempsey 2006). The supergiant species are *B.crosnieri* Lowry & Dempsey 2006, *B.giganteus* Milne Edwards, 1879, *B.jamesi* Kou, Chen & Li, 2017, *B.keablei* Lowry & Dempsey, 2006, *B.kensleyi* Lowry & Dempsey, 2006 *B.lowryi* Bruce & Bussawarit, 2004, *B.raksasa* Sidabalok, Wong & Ng, 2020, *B.richeri* Lowry & Dempsey, 2006, and *B.yucatanensis* Huang, Kawai & Bruce, 2022.

**Figure 1.**
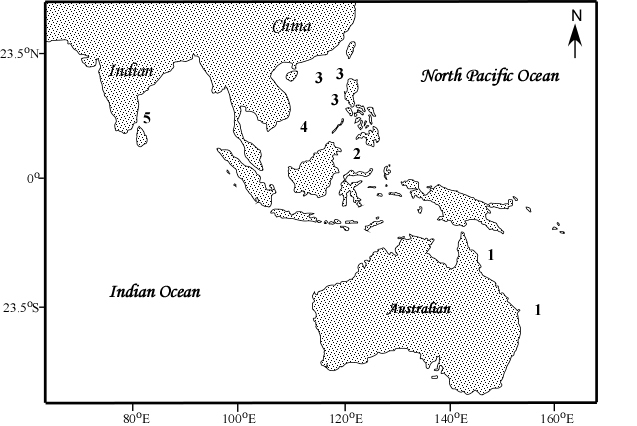
Map of specimens identified as *Bathynomuskensleyi*. 1. *B.kensleyi* Lowry and Dempsey 2006 *sensu strictu* off the Great Barrier Reef, eastern Australia; 2. *B.kensleyi* part Lowry and Dempsey (2006); Sulu Sea (= *Bathynomus* sp., undescribed); 3. South China Sea, off Hong Kong, Taiwan and Pratas Island (Lowry and Dempsey 2006) (= *B.jamesi*); 4. off Spratly Island (Truong 2015) (= *Bathynomus* sp); and 5. off Parangipettai, south-eastern India (Sankar et al. 2011, PrasannaKumar et al. 2020) (=*Bathynomus* sp).

**Figure 2.**
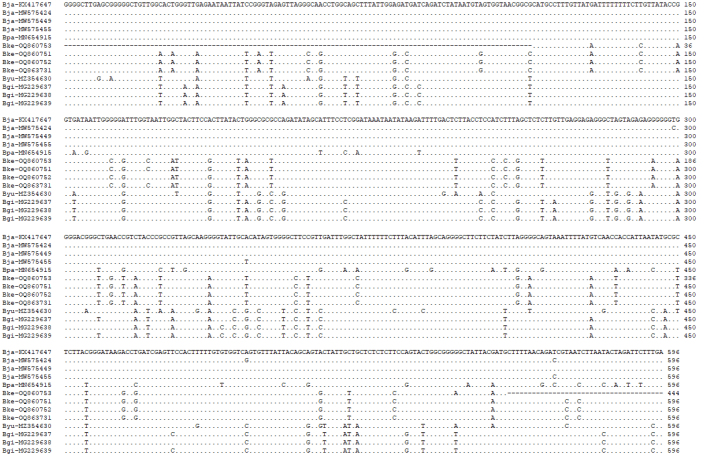
Alignment of the partial DNA sequence of the cytochrome *c* oxidase I from several supergiant *Bathynomus* spp, *B.jamesi* (Bja, NCBI Acc. Nos. KX417647, MW575424, MW575449, MW575455), B.cf.keablei. (Bpa, MN654915 (from Parangipettai)), *B.kensleyi* (Bke, OQ860751, OQ860752, OQ863731 and holotype OQ860753), *B.yucatanensis* (Byu, MZ354630) and *B.giganteus* (Bgi, MG229637, MG229638 and MG229639).

Most recently, some records of *Bathynomus*, if correct, would have shown vast range extensions for three species from the western Pacific to the northern Indian Ocean. The species are *Bathynomusdecemspinosus* Shih, 1972, *Bathynomusdoederleini* Ortmann, 1894 and *Bathynomuskensleyi* Lowry & Dempsey, 2006 recorded from India by Sankar et al. (2011), those identifications later being repeated by PrasannaKumar et al. (2020). Their identification, however, maybe incorrect. Two of the species, *B.decemspinosus* and *B.doederleini* are ‘giants’ of less than 15 cm body length, while all the illustrated figures and measurements, given by Sankar et al. (2011) and PrasannaKumar et al. (2020), show that their specimens are ‘supergiants’ of greater than 17 cm body length (22 to 33 cm); further, the uropod details (Sankar et al. 2011. figs. 1 and 2; PrasannaKumar et al. 2020, fig. 1) are wholly incompatible with illustrations for those two species (Bruce 1986, figs. 87F–K 88G–I; Lowry and Dempsey 2006 figs. 10 and 11).

The identity and identification of Indian records of *B.kensleyi* is more ambiguous. The pleotelson and uropods of the Indian species and *B.kensleyi* are similar and, while the photos lack adequate detail, the uropodal apices of all the Indian figured specimens align more with *B.keablei* rather than *B.kensleyi* in that there is no evident distolateral point on the uropodal rami. All the Indian specimens figured appear to be one species and the closest species would appear to be *Bathynomuskeablei* Lowry and Dempey 2006, a ‘supergiant’ that is known from Indian waters. It is not possible to definitively re-identify these from the figures in Sankar et al. (2011) and PrasannaKumar et al. (2020) as *B.keablei*, though we note that *B.keablei* was recorded from both the eastern and western coasts of India, as well as off Myanmar at depths of 400 to 1353 m (Lowry and Dempsey 2006). Fifty-one specimens of *Bathynomus* from Parangipettai were examined by Sankar et al. (2011) and vouchers deposited at the Museum of the Centre of Advanced Study in Marine Biology, Annamalai University, Parangipettai, Tamil Nadu, India, so it should be possible, at some point, for all these specimens be examined in detail and their correct identity established.

Notwithstanding the morphological issues regarding *Bathynomus* taxonomy, new molecular data presented here unambiguously demonstrate that the Indian specimens are not *Bathynomuskensleyi*, confirming the opinion of Huang et al. (2022).

Based on the above issues, gene sequencing and research of *B.kensleyi* are the keys to solving the problem of identity of these similar species. Amongst them, the COI sequence becomes the most critical classification basis. In this study, the tissues of *B.kensleyi* holotype (NTM Cr003425) were obtained from the Museum and Art Gallery of the Northern Territory in Australia. In addition, the muscle tissues of three new samples (sample numbers W29628, W29629 and W29630) were obtained with the help of the Queensland Museum, the COI sequence of *B.kensleyi* was successfully analysed by molecular biology methods and the above-mentioned question of whether *B.jamesi* and *B.kensleyi* were the same species, based on morphological data (Huang et al. 2022), is here resolved using molecular data that clearly show the two species are distinct.

## Materials and Methods

### Specimen collection

The experimental samples were *B.kensleyi* holotype (NTM Cr003425) pereopod muscle tissue (in 70% ethanol) provided by the Museum and Art Gallery of the Northern Territory and three recently-collected *B.kensleyi* (W29628, W29629 and W29630) pereopod muscle tissue were impregnated with high-grade ethanol by the Queensland Museum. After the samples arrived at the laboratory, they were stored in a -20°C refrigerator until needed for the experiment.

The collection data of *B.kensleyi* holotype are as follows: Northern Territory Museum Cr003425, Marion Plateau, Coral Sea, QLD, Australia (22.9167°S, 154.3501°E, depth, 590–606 m, Stn: 0685–08, coll: NL Bruce, 17 November 1985, det: J. Lowry 2004. (Fig. 1)

The data of three new specimens of *B.kensleyi* are as follows: Queensland Museum *B.kensleyi* W29628, W29629 and W29630 were collected at the same time, place and collector. East of Heron Island, MEQ (-23.2532, 153.8718), 700–800 m depth, Nov 2022, coll: David Hand, det: NL Bruce.

To facilitate discussion, the species from south-eastern India, misidentified as *B.kensleyi*, *B.doederleini* and *B.decempinosus*, are collectively referred to as Bathynomus ' cf. keablei' (see 'Background' in the introduction).

### Molecular analysis

Total genomic DNA was extracted from ca. 25 mg each of pereopod muscle harvested from all specimens from Australian material, using a commercial genomic DNA extraction kit (QIAamp DNA Mini Kit, Hilden, Germany) according to the manufacturer’s protocol. PCR primers (LCO-1490 and HCO-2198) used for the amplification were designed, based on the sequences of the genes encoding COI (Folmer et al. 1994) and 16S ribosomal RNA (Palumbi et al. 1991) of *B.kensleyi* (Table 1). In addition, using the COI sequence confirms primers as TESCOI for double-checking (Table 1). All samples (holotype NTM Cr003425, W29628, W29629 and W29630) were sequenced for COI and 16S rRNA.

Amplification using the COI and 16S rRNA primers was based on a cycle of denaturation at 94ºC for 30 s, annealing at 48ºC for 40 s and extension at 72ºC for 30 s using a DNA thermal cycler model MyCycler^TM^ Thermal Cycler System (#1709703, Bio-Rad, Hercules, CA, USA). This procedure was carried out for 35 cycles and the final extension step was performed at 72ºC for 10 min. The 100 μl reaction medium contained 200 nM dNTPs, 10 mM each of forward and reverse primers, two units of Ex-Tag DNA polymerase (TaKaRa Ex Taq^®^ DNA Polymerase, Takara Bio, Shiga, Japan), 10 μl of 2×Ex-Tag DNA polymerase buffer (Takara Bio) and 50 ng of genomic DNA. The PCR products were subjected to electrophoresis using 2% agar (VWR Funding Inc, West Chester, PA, USA) and visualised with Nucleic Acid Stain (HealthView^TM^, Genomics, Xizhi District, New Taipei City, Taiwan). After confirming the success of PCR amplification, the products were sent to a biotech company (Genomics, Xizhi District, New Taipei City, Taiwan) for sequencing. The obtained sequences were edited and aligned using editing software BioEdit 7.2 (https://www.mybiosoftware.com/bioedit-7-0-9-biological-sequence-alignment-editor.html) and Multiple Sequence Alignment (Clustal Omega – GenomeNet, Hinxton, Cambridgeshire, UK).

During the experiment, primers LCO-1490 and HCO-2198 were used in the PCR process at the beginning, but the PCR results smeared seriously, showing a non-specific increase in PCR, which reflected the lack of specificity of the primers LCO-1490 and HCO-2198. To increase the specificity of the primer, methods of increasing the temperature and redesigning the primer were tried. Trials using 40ºC (Folmer et al. 1994), 48ºC (Kou et al. 2017, Huang et al. 2022) and 54ºC (PrasannaKumar et al. 2020), finally confirmed that 48ºC is the best increase in *B.kensleyi* COI temperature. Due to the smear phenomenon after PCR, primers such as TESCOI(F), TESCOI(R), KensMae(F) and KensMae(R) were replaced successively and forward and reverse primers were used crosswise and finally a complete DNA sequence was obtained.

### Kimura 2-parameter distance

Comparisons of the edited and aligned COI and/or 16S rRNA sequences of the present specimens and five supergiant previously sequenced species of *Bathynomus* were performed using the Molecular Evolutionary Genetics Analysis 11 (MEGA 11) software (Tamura et al. 2021). COI sequence data were obtained from the National Center for Biotechnical Information (NCBI) for *B.giganteus* (NCBI Acc. Nos. MG229637, MG229638 and MG229639) (from the northern Gulf of Mexico, except De Soto Canyon, Timm et al. (2018), *B.jamesi* (KX417647, holotype, from the sea off the southern part of Hainan Island, China, Kou et al. (2017)), (MW575424, MW575449 and MW575455) (from the sea of Pratas Island and the South China Sea), *B.yucatanensis* (MZ354630, holotype from the Gulf of Mexico off the Yucatan Peninsula, Huang et al. (2022)), B.cf.keablei (DBGI1, MN654914), B.cf.keablei (DBGI2, MN654915) and B.cf.keablei (DBGI3, MN654916 (misidentified by PrasannaKumar et al. (2020) as *B.kensleyi*, *B.decemspinosus* and *B.doederleini* from the coast of Parangipettai, India, PrasannaKumar et al. (2020))). 16S rRNA sequences for *B.jamesi* (KX417641, KX417643 and MZ029589) (from the sea off the southern part of Hainan Island, China, Kou et al. (2017)), *B.giganteus* (MG229477, MG229478 and MG229479) (from the northern Gulf of Mexico, except for De Soto Canyon Timm et al. (2018)) and *B.yucatanensis* (MZ042927, holotype) were obtained (Table 2) .

This study lists all supergiant *Bathynomus* COI sequence analyses registered in NCBI. Therefore, an external control was added as an analysis (Horiike 2016). The nucleotide sequence for Cirolanidae COI (*Atarbolanaexoconta* Bruce and Javed 1987, KX782999) and 16S rRNA (*Excirolanahirsuticauda* Menzies 1962, MK898194) were used as the outgroup control, respectively. Using Drawtree (Phylip software package, http://bioweb.pasteur.fr/seqanal/interfaces/drawtree.html), molecular trees were constructed by the neighbour-joining (NJ) method under the Kimura 2-parameters (K2P) distance (Kimura 1980). Using K2P distance in MEGA 11, pair-wise distance analysis was carried out (Tamura et al. 2007).

## Results

### Sequence of new samples of B.kensleyi W29628, W29629 and W29630

**Figure 3.**
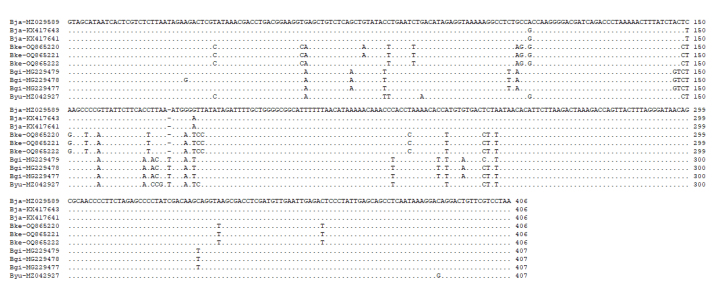
Alignment of the partial DNA sequence of the 16S rRNA from several supergiant *Bathynomus* spp, *B.jamesi* (Bja, NCBI Acc. Nos. KX417641, KX417643 and MZ029689), *B.kensleyi* (Bke, OQ865220, OQ865221 and OQ865222), *B.yucatanensis* (Byu, MZ042927) and *B.giganteus* (Bgi, MG229477, MG229478 and MG229479).

The primers LCO-1490 and HCO-2198 (Table 1) were initially tested for the DNA sequence of the COI for *B.kensleyi* gene cloning. The first successful sample attempt to increase was W29629—amplified PCR products of 681 bp from COI. Due to the severe smear bands when using primers LCO-1490 and HCO-2198, samples W29628 and W29630 did not complete PCR amplification smoothly. Due to the failure of PCR amplification, TESCOI(F), TESCOI(R), KensMae(F) and KensMae(R) (Table 1) were tried. Finally, all three successfully resolved the COI sequence and the COI sequences of W29628, W29629 and W29630 each obtained 681 bp. Fig. 2 lists 596 bp (the shorter holotype) in the sequence as an alignment with other species. It can be seen from Fig. 2 that the sequences of *B.kensleyi* are almost identical. The COI sequences of W29628, W29629 and W29630 have been uploaded to DDBJ/EMBL/GenBank (Acc. Nos. OQ860751, OQ860752 and OQ863731, respectively). As another marker, the sequence of 16S rRNA was also resolved successfully. 16S rRNA PCR amplification uses 16SarF and 16SbrR (Table 1) as primers and a 514 bp DNA sequence (Fig. 3) is obtained. The DDBJ/EMBL/GenBank Acc. Nos. were OQ865220 (W29630), OQ865221 (W29628) and OQ865222 (W29629). In the 16S rRNA sequence, the 16S rRNA of three (W29628, W29629 and W29630) new samples had only one nucleotide difference (ca. 73, A > G) (Fig. 3)

#### Sequence of *B.kensleyi* Holotype NTM Cr003425

The *B.kensleyi* holotype (NTM Cr003425) provided by the Museum and Art Gallery of the Northern Territory, which had been in alcohol for more than 37 years, was initially unable to be amplified in the PCR reaction, causing the experiment to be suspended for several months. After obtaining new samples from the Queensland Museum and successfully obtaining the 681 sequences of COI, the *B.kensleyi*-specific primers (KensMae(F) and KensMae(R)) were redesigned and amplified in the PCR reaction. A total of 444 bp of COI was obtained after the PCR amplification product was sequenced (Fig. 2). Although not all of the 681 bp of new samples, 444 bp could be used for species comparison. After DNA comparison, it was found that the sequence was almost identical to the new samples W29628, W29629 and W29630, except for one nucleotide (Fig. 2, ca. 546, G > A). This variation may be single nucleotide polymorphisms (SNPs) (Syvänen 2001) (Fig. 2). The DNA sequence of *B.kensleyi* holotype has been uploaded to DDBJ/EMBL/GenBank (Acc. No. OQ860753). Possibly, the DNA content of the specimen was too low or the specificity of the primer was not specific enough and the PCR amplification of 16S rRNA was unsuccessful. The alignment of the partial DNA sequence of the 16S rRNA from several supergiant *Bathynomus* is shown in Fig. 3.

### Molecular analysis

**Figure 4.**
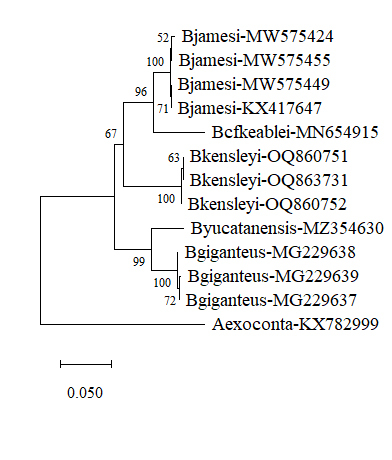
Molecular tree, based on the DNA sequences of cytochrome *c* oxidase I (COI). The sequences were aligned using Clustal Omega and the tree was constructed by the neighbour-joining method. Numbers at branches indicate bootstrap values. The sequences of Cirolanidae (*Atarbolanaexoconta*, KX782999) COI were used as the outgroup. Evolutionary analyses were conducted in MEGA 11. *B.jamesi* (NCBI Acc. Nos. KX417647, MW575424, MW575449 and MW575455), B.cf.keablei. (MN654915 (from Parangipettai)), *B.kensleyi* (OQ860751, OQ860752 and OQ863731), *B.yucatanensis* (MZ354630) and *B.giganteus* (MG229637, MG229638 and MG229639).

**Figure 5.**
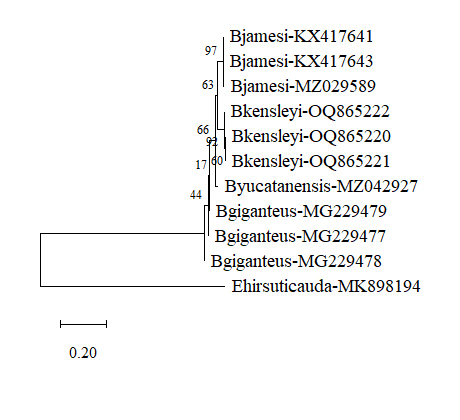
Molecular tree, based on the DNA sequences of 16S rRNA. The sequences were aligned using Clustal Omega and the neighbour-joining method constructed the tree. Numbers at branches indicate bootstrap values. The sequences of Cirolanidae (*Excirolanahirsuticauda* Menzies, 1962, MK898194) 16S RNA was used as the outgroup. Evolutionary analyses were conducted in MEGA 11. *B.jamesi* (NCBI Acc. Nos. KX417641, KX417643 and MZ029589), *B.kensleyi* (OQ865220, OQ865221 and OQ865222), *B.yucatanensis* (MZ042927) and *B.giganteus* (MG229477, MG229478 and MG229479.

Our analysis is based on the new *B.kensleyi* COI DNA sequence and other known supergiant *Bathynomus* (only four of nine supergiant species have been registered on DDBJ/EMBL/GenBank database) sequences being from *B.jamesi*, *B.giganteus*, *B.kensleyi*, *B.yucatanensis* and B.cf.keablei (DBGI2, misidentified of *B.kensleyi* PrasannaKumar et al. (2020)), *Atarbolanaexoconta* (KX782999) being used as an external control. Using MEGA 11, the evolution tree derived by neighbour-joining method is shown in Fig. 4. The same species form a cluster, revealing the relative relationship. In addition, molecular analysis, based on 16S rRNA, was also carried out and *Excirolanahirsuticauda*, MK898194 was used as the external control. The results are shown in Fig. 5.

### Kimura 2-parameter distance

To compare inter-species and intra-species variability, the Kimura 2-parameter (K2P) distance (Kimura 1980) for the COI gene was used to compare *B.kensleyi* and *B.jamsie*. Based on the K2P distance, the average inter-specific distance (11.48%) was 39-fold higher than the average intra-specific distance (0.29%) (Table 3). There was a clear-cut barcode gap (5.81%–17%) between the maximum intra-specific distance. On the other hand, the average inter-specific distance (6.07%) was 18-fold higher than the average intra-specific distance (0.33%) for the 16S rRNA gene (Table 4). There was a clear-cut barcode gap (4.08%–7.35%) between the maximum intra-specific distance.

The other two reported Indian species of B.cf.keablei (misidentified by PrasannaKumar et al. (2020) as *B.decemspinosus* DBGI1, MN654914) and B.cf.keablei (misidentified by PrasannaKumar et al. (2020) as *B.doederleini*, DBGI3, MN654916) COI genes have also been checked. In the species of B.cf.keablei (misidentified of *B.doederleini*, DBGI3) , the average inter-specific distance (1.08%) (references were used NCBI database and DDBJ/EMBL/GenBank numbers as follows: MZ723938, MZ723939, MZ726388, OQ913469, AB851912 and OQ421549) was 4.5-fold higher than the average intra-specific distance (0.24%) for COI gene (data not shown). There was a clear-cut barcode gap (0.95%–6.5%) between the maximum intra-specific distances. The species *B.decemspinosus* has no holotype sequence registered in the NCBI database, so its comparisons cannot be made and previous inferences are questionable; at present, *B.decemspinosus* can only be identified using morphological characters.

## Discussion

Reliable species identification techniques and methods are necessary to conserve, manage and sustainably develop natural resources. Morphological taxonomy is a valuable tool for identifying species and has stood the test of time, but by itself, is not always reliable and morphological taxonomy cannot always be used to identify some cryptic species. Morphology refers to the physical characteristics of an organism, including its size, shape and other visible features. Through morphological identification, the characteristics and correlations of organisms can be distinguished.

### Genetics and species identification

Genetic analysis is already proving highly useful in distinguishing and identifying species of *Bathynomus* (Huang et al. 2022). As the overall appearance of some species within the two groups of ‘giants’ and ‘supergiants’ may appear almost the same, it adds to the difficulty in identifying both described and undescribed species. DNA sequences of highly-conserved genes, such as the COI gene, have been used to identify biological species. Current evidence shows that COI identification works well, especially for species with a slight morphological variation or biological species that retain only a portion of their tissues (Lobo et al. 2013; Elbrecht et al. 2016). Hebert et al. (2003) suggested that a DNA barcode could be the most helpful tool for identifying biological species. The 16S rRNA is another commonly-used biomarker (Vences et al. 2005) and a few sequences of this gene have been recorded for *Bathynomus* spp. in recent years (Kou et al. 2017, Timm et al. 2018, Huang et al. 2022).

The advantage of using genetics, such as COI and 16S rRNA as markers to identify species, is their high level of accuracy. When DNA sequences are compared, it is easy to see whether or not they are the same species and easy to understand, even without using statistics or K2P. However, individual differences lead to a small amount of DNA variation called single nucleotide polymorphisms (SNPs). According to research by *Bathynomus*, the probability of SNPs appearing in gene COI is low. Take *B.jamesi* as an example; in DNA sequences with a known length of about 600 bp, there are rarely more than five SNPs and the most common number of SNPs is 0-3 (Huang et al. 2022). In addition, there are drawbacks; these DNA sequences are useless when a species is misidentified. This erroneous and misleading information may be repeatedly cited and even seriously affect the direction of follow-up research (Huang et al. 2022).

Species of *Bathynomus* are not only very similar in overall shape, but the appendages are also often generally similar in appearance and species are generally not easy to distinguish by morphological appearance. There is also some slight intra-specific variation within the same species of *Bathynomus*. In addition, the number of specimens and species researched is low and it is not easy to compare individuals. Based on the above reasons, it is often difficult for species of *Bathynomus* to be identified and, where differences are observed, there may be some uncertainty over whether the differences belong to intra-species or inter-species variation. Morphology remains the standard for biological identification, but as more species of *Bathynomus* are described and redefined, four species (*B.jamesi*, *B.maxeyorum*, *B.raksasa* and *B.yucatanensis*) have been identified since the taxonomic key of Lowry and Dempsey (2006) and that, crucially, already needs to be revised. As morphological species detection becomes ever finer, it seems inevitable that the time will come when species in the genus may be determined solely by molecular data.

### Analysis of B.kensleyi holotype

The distribution of *Bathynomuskensleyi* was regarded by Lowry and Dempsey (2006) to extend from eastern Australia to the Philippines and the South China Sea. Sankar et al. (2011) developed the purported range to the northern Indian Ocean. Misidentifications of *B.kensleyi* have led to illogical results in subsequent research on *Bathynomus*. For those reasons, this study uses COI as the primary marker to distinguish the similarities and differences between the sequences of *B.jamesi* and *B.kensleyi*, as a basis to determine whether or not they are the same species. In addition to COI, 16S rRNA is also used as a marker to compare the similarities and differences between *B.jamesi* and *B.kensleyi*.

After obtaining the *B.kensleyi* PCR amplification conditions, we again tried to sequence tissue from the holotype of *B.kensleyi* (NTM Cr003425). Extraction from the *B.kensleyi* holotype failed as the muscles, most of which are fascia and other pereopod tissue, were decomposed and the concentration of DNA too low. After several failed attempts, the COI gene was successfully amplified by PCR using newly-designed primers (KensMae(F) and KensMae(R)) with higher specificity and obtained part (444 bp) of the COI DNA sequence. After comparing this holotype COI sequence with three new samples, it was confirmed that the four (holotype +3 new samples) belonged to the same species (Fig. 2).

According to the DNA sequence alignment (Fig. 2), the COI sequence structures of three new *B.kensleyi* (OQ860751, OQ860752,and OQ863731) have a high degree of identity. Only one of the 596 DNA sequences differed (OQ860752, ca. 171, C > A, Fig. 2). The data showed that the three were the same species. Different nucleotides show inter-individual differences and non-systematic differences can be considered single nucleotide polymorphism in individuals of the same species (Huang et al. 2022).

In addition, comparing *B.jamesi* COI sequences (KX417647, MW575424, MW575449 and MW575455) (Fig. 2), it can be found that the DNA sequence differences between *B.jamesi* and *B.kensleyi* are relatively high. Amongst the 596 DNA sequence comparisons, 59 bases differ (MW575424 vs. OQ860751) (Fig. 2) and the DNA sequence similarity is 90.1%.

### Kimura 2-parameter distance

The K2P distance is a tool for quantifying and comparing the variability of two gene sequences (Zhang and Hanner 2011). To test the degree of DNA sequence divergence between *B.jamesi* and *B.kensleyi*, the K2P distance was introduced as a tool for inter-species and intra-species analysis. As shown in Table 3, the K2P distance of COI between *B.jamesi* and *B.kensleyi* ranged from 10.01% to 10.82%, with an average value of 10.29%, which was far greater than the average value of the intra-specific variation of 0.29% (35-fold higher). *B.jamesi* and *B.kensleyi* belong to two different species (Zhang et al. 2021).

The same method (K2P) was used to test whether the “*B.kensleyi*” (B.cf.keablei) in the Indian waters, referred to by PrasannaKumar et al. (2020), is the same as the *B.kensleyi* from Australia. The *B.kensleyi* COI sequence (OQ860751, OQ860752 and OQ863731) has more than 85 DNA nucleotides differences with B.cf.keablei (MN654915, Fig. 2) and the value of K2P distance was 14.4% - 14.62% (Table 3), which was significantly higher than the inter-specific distance (0.29%), showing it to be different from *B.kensleyi*. This result confirms the hypothesis of Huang et al. (2022) that the supergiant *Bathynomus*, B.cf.keablei, from Indian Parangipettai is misidentified. The molecular analysis of this species shows that the closest relative is *B.jamesi* (Fig. 4, Table 3).

PrasannaKumar et al. (2020) also used K2P to detect distances between inter-species and intra-species. The data show that the K2P distance of *B.jamesi* (KX417646) and *B.giganteus* (KT963284) is 0.12 (PrasannaKumar et al. 2020, p.3, Table 2). These data are the same value as the K2P distance in Table 3 *B.jamesi* (MW575424) and *B.giganteus* (MG229639) in this paper. Both papers use the same calculation method for analysis. However, observing the other values in Table 2 of PrasannaKumar et al. (2020), they are greater than the average value of the intra-species variation calculated in this paper, 0.29%, so it is inferred that the identities of the species referred to by PrasannaKumar et al. (2020) need to be re-examined. Further, Kou et al. (2017) and Huang et al. (2022), in sequencing *B.kensleyi*, obtained the PCR product gain at an annealing temperature of 48°C. The same primers (LCO-1490, HCO-2198) were used by PrasannaKumar et al. (2020), but with an annealing temperature 6°C higher than the standard 48°C (54°C was used for annealing temperature by PrasannaKumar et al. (2020)). As the PCR reaction is a susceptible chemical reaction, a difference in temperature of this magnitude could plausibly affect the gains of the PCR product and the two results may not be entirely comparable.

### Kimura 2-parameter distance reflects geographical distribution

In addition, using the K2P analysis of COI revealed an interesting phenomenon - the value reflects the distance of geographical distribution. For example, the minimum value of K2P distance appeared in *B.yucatanensis* vs. *B.giganteus* (5.81% - 6.19%) (Table 3), followed by B.cf.keablei vs. *B.jamesi* (6.88% - 7.07%), reflecting that the geographical distribution of *B.yucatanensis* vs. *B.giganteus* is close and also that the Bay of Bengal (B.cf.keablei) and the South China Sea (*B.jamesi*) is also close. On the other hand, the maximum K2P distance of COI appeared in *B.yucatanensis* vs. B.cf.keablei (17%), followed by *B.giganteus* vs. B.cf.keablei (15.49% - 15.71%) which also reasonably reflects the geographical distribution.

This analysis found that using COI as a marker can more faithfully reflect the facts than 16S rRNA as a marker. It may be one of the reasons why COI is widely used as a DNA barcode (Table 3, Table 4).

The molecular tree was drawn using MEGA 11 (Fig. 4). The molecular relationship and geographical relationship of the species of *Bathynomus* are shown in the COI molecular tree (Fig. 4). For example, *B.giganteus* and *B.yucatanensis* are closely related, while *B.jamesi* and B.cf.keablei are relatively close. Therefore, it is reasonable that geographic relatedness is also reflected in the molecular trees.

### Review of three types of Bathynomus record from India

Sankar et al. (2011) and PrasannaKumar et al. (2020) referred to three species of *Bathynomus* found in the Indian Ocean, namely *B.kensleyi*, *B.doederleini* and *B.decemspinosus*. Amongst those species, *B.doederleini* and *B.decemspinosus* belong to the giant species and the body length should be less than 15 cm (Lowry and Dempsey 2006). The sizes of the specimen in the figures provided by Sankar et al. (2011) (p144, figs. 1 and 2) suggest that the identifications are incorrect. However, molecular data can also be used to analyse differences between *B.kensleyi* and *B.doederleini* and the results obtained are not the cited species. We conclude that, at present, there is only one authoritatively named species of *Bathynomis*, *B.keablei*, known from Indian waters.

## Conclusions

Finally, we make a summary. In this study, using the *B.kensleyi* samples provided by the Museum and Art Gallery of the Northern Territory and the Queensland Museum in Australia, it was confirmed that *B.kensleyi* and *B.jamesi* are different species through COI and 16S rRNA sequences. The notion that *B.kensleyi* and *B.jamesi* are the same species is refuted.

## Figures and Tables

**Table 1.** List of primer pairs and PCR annealing temperatures (*T*m) used to amplify COI and 16S rRNA genes.

Primers	Sequence 5'-3'	*T*m (^O^C)
COI primers (Folmer et al. 1994):		
LCO-1490 (F)	GGT CAA CAA ATC ATA AAG ATA TTG G	48
HCO-2198 (R)	TAA ACT TCA GGG TGA CCA AAA AAT CA	48
TESCOI (F)	TAG TGG TAA CGG CTC ATC CC	53
TESCOI (R)	GCA TTG TAA TAG CTC CCG CC	53
KensMae (F)	GTT GGA CA GGG TTA AGA AT	48
KensMae (R)	AGT ATT AAG GTT GCG ATC TG	48
16S primers (Palumbi et al. 1991):		
16Sar (F)	CGC CTG TTT ATC AAA AAC AT	43
16Sbr (R)	CCG GTC TGA ACT CAG ATC ACG T	43

**Table 2.** *Bathynomus* species, accession numbers of the National Center for Biotechnical Information and references.

Species/genes	NCBI Acc.Nos.	References
COI		
*B.jamesi*	KX417647, MW575424, MW575449, and MW575455	Kou et al. (2017), Huang et al. (2022)
*B.giganteus*	MG229637, MG229638, and MG229639	Timm et al. (2018)
*B.kensleyi*	OQ860751, OQ860752, OQ863731, and OQ860753	This paper
*B.yucatanensis*	MZ354630	Huang et al. (2022)
B.cf.keablei	MN654914, MN654915, and MN654916	PrasannaKumar et al. (2020)
16S rRNA		
*B.jamesi*	KX417641, KX417643, and MZ029589	Kou et al. (2017), Huang et al. (2022)
*B.giganteus*	MG229477, MG229478, and MG229479	Timm et al. (2018)
*B.kensleyi*	OQ865220, OQ865221, and OQ865222	This paper
*B.yucatanensis*	MZ042927	Huang et al. (2022)

**Table 3.** The pairwise distance (K2P distance) of COI gene segment (596 bp) amongst studied species of *Bathynomus*. Numbers in italics indicate intra-specific divergence. Numbers in parentheses indicate the number of individuals.

	1	2	3	4	5
*B.jamesi* (4)	*0-0.0051*				
B.cf.keablei	0.0688-0.0707	0			
*B.kensleyi* (3)	0.1001-0.1082	0.1440-0.1462	*0-0.0017*		
*B.yucatanensis*	0.1248-0.1290	0.17	0.1265-0.1287	0	
*B.giganteus* (3)	0.1126-0.1209	0.1549-0.1571	0.1248-0.1290	0.0581-0.0619	*00017-0.0051*

**Table 4.** The pairwise distance (K2P distance) of 16S rRNA gene segment (406-407 bp) amongst studied species of *Bathynomus*. Numbers in italics indicate intra-specific divergence. Numbers in parentheses indicate a number of individuals.

	1	2	3	4
*B.jamesi* (3)	*0-0.0074*			
*B.kensleyi* (3)	0.0595-0.0678	*0-0.0025*		
*B.yucatanensis*	0.0513-0.0567	0.0487-0.0514	0	
*B.giganteus* (3)	0.0567-0.0622	0.0679-0.0735	0.0408-0.0434	*0.0025-0.0049*
